# The ACP-ASC model: A comprehensive behaviour change model for advance care planning based on the Stages of Change model

**DOI:** 10.1177/02692163261431148

**Published:** 2026-03-29

**Authors:** Tessa D. Bergman, Bregje D. Onwuteaka-Philipsen, Eva E. Bolt, H. Roeline W. Pasman, Annicka G. M. van der Plas

**Affiliations:** 1 Department of Public and Occupational Health, Amsterdam Public Health Research Institute, Expertise Centre for Palliative Care, Amsterdam UMC, Vrije Universiteit Amsterdam, The Netherlands; 2 Department of General Practice, Amsterdam Public Health Research Institute, Expertise Centre for Palliative Care, Amsterdam UMC, Vrije Universiteit Amsterdam, The Netherlands

**Keywords:** advance care planning, behaviour change, transtheoretical model, Stages of Change, end-of-life care

## Abstract

**Background:**

To design and implement interventions that promote advance care planning, behaviour change models can be useful. A widely used model is the Transtheoretical model by Prochaska and DiClemente (1982), describing five Stages of Change: precontemplation, contemplation, preparation, action, and maintenance. However, this model does not fully accommodate the advance care planning process. First, advance care planning can include not one, but ideally up to three actions: discussing wishes with family/friends, with healthcare professionals, and documentation. Second, the order of these actions can vary, and each action can be omitted. Third, advance care planning is an ongoing process in which individuals ideally reflect upon their wishes regularly; therefore, maintenance should be considered as part of action.

**Aim:**

To develop a comprehensive behaviour change model for advance care planning based on the Stages of Change: the Advance Care Planning–Applied Stages of Change model (ACP-ASC model).

**Methods:**

The ACP-ASC model is based on current literature and data from a nationwide sample of older people. It was conceptualized through extensive discussions within a multidisciplinary research team, including healthcare professionals.

**Insights:**

To accommodate the complexity of advance care planning, the action stage of the ACP-ASC model distinguishes three types of advance care planning actions (discussions with family/friends, with healthcare professionals, and documentation) and does not prescribe an order. Further, maintenance is incorporated in the action stage, resulting in levels of action (first; maintained) for each type of advance care planning action. Last, we discuss further considerations, practice implications, and recommendations for future research.

**What is already known about the topic?**

- Over time, advance care planning has evolved from the completion of an advance directive to an ongoing communication process with family, friends and healthcare professionals about end-of-life preferences.
- The Stages of Change of the Transtheoretical model is commonly used to guide behaviour change but does not fully accommodate the complexity of the ongoing advance care planning process with multiple interacting actions.

**What this paper adds**

- This paper introduces the Advance Care Planning–Applied Stages of Change model (ACP-ASC model): a comprehensive behaviour change model for advance care planning based on the Stages of Change.
- The ACP-ASC model differentiates three distinct advance care planning actions—discussions with family/friends, with healthcare professionals, and documentation—without prescribing a fixed order.
- The ACP-ASC model integrates maintenance into the action stage, recognizing advance care planning as an ongoing and iterative process.

**Implications for practice, theory, or policy**

- The ACP-ASC model provides a more nuanced understanding of advance care planning engagement, supporting tailored interventions across diverse populations.
- It offers a practical tool for clinicians and policymakers to assess and promote advance care planning readiness and engagement.
- The model lays a foundation for future research to evaluate and refine advance care planning interventions based on behavioural stage progression.

## Introduction

Over time, advance care planning has evolved from the completion of an advance directive to a communication process with family, friends and healthcare professionals about end-of-life preferences.^1–3^ The role of an advance directive changed from the main aim to an optional element of advance care planning. Today, advance care planning is considered an ongoing process of reflection, clarification, and communication of values.^4^ The 2017 EAPC-supported international consensus definition states that ‘advance care planning is a process that enables individuals to define goals and preferences for future medical treatment and care, to discuss these goals and preferences with family and healthcare professionals, and to record and review these preferences if appropriate’.^3^ Individuals can engage in advance care planning in any stage of their life but the content discussed can be more specific as their health condition worsens or as they age.^3^

**Figure 1.**
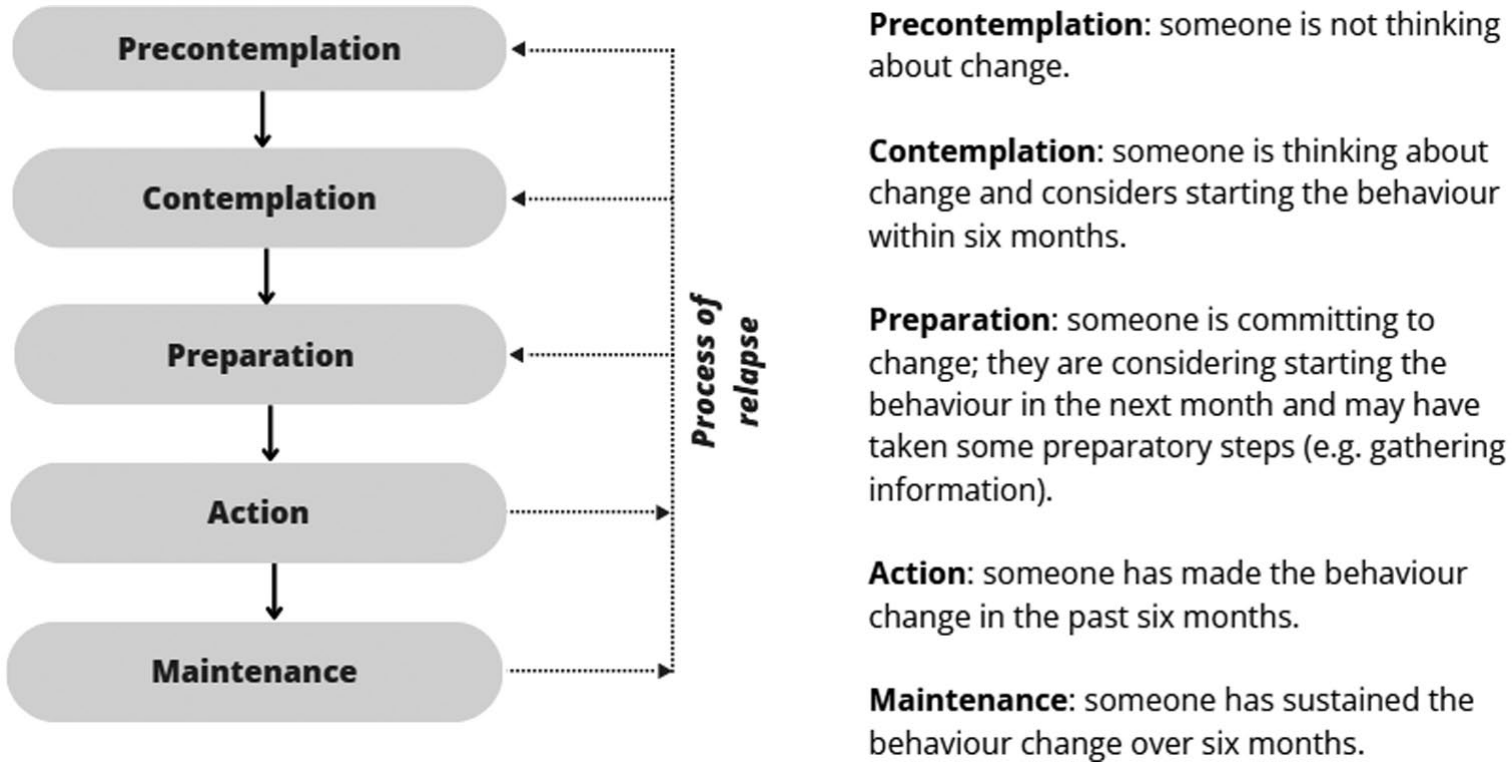
The Stages of Change model by Prochaska and DiClemente. (Prochaska and DiClemente, 1982)(Prochaska and DiClemente, 1983)(Prochaska and Norcross, 2018)

Extensive research is available on the development and effectiveness of interventions designed to stimulate advance care planning.^5^ Advance care planning is a behaviour, but behavioural theories are rarely applied to advance care planning.^6^ Behavioural theories aim to systematically identify antecedents to health behaviour and aid to develop and evaluate interventions,^7^ and can increase effectiveness.^6,8^ The Transtheoretical model by Prochaska and DiClemente is a widely used behaviour change theory. It includes the Stages of Change model, which consists of five stages based on individuals’ current behavioural status and intention to change (Figure 1).^9–11^

The Stages of Change model has been used in previous advance care planning (intervention) research to describe empirical studies on engagement in advance care planning,^6,8,12–26^ often focussing on elements of advance care planning such as advance directive completion.^14,16,20,26^ Stage-matched interventions are interventions tailored to the appropriate processes of change at each Stage of Change of the Transtheoretical model. Such enhance both the acceptability and effectiveness of interventions, thereby supporting behaviour change.^8,27^ However, the original Stages of Change model does not fully accommodate the advance care planning process as it is understood today, given its complexity as an ongoing process with multiple interacting actions. Authors struggle with the use of the model in advance care planning. For example, Fried et al.^18^ described advance care planning discussions with family/friends, with healthcare professionals, and the completion of an advance directive as separate behaviours, resulting in multiple Stages of Change models. However, these three actions are interconnected within the broader advance care planning process and do not function in isolation. Additionally, Ernecoff et al.^15^ refers to a combined ‘action-maintenance stage’, without further reflection. Moreover, a systematic review of advance care planning interventions concluded that little is known about (the interpretation of) the maintenance stage in advance care planning, stressing the need for further research.^8^

The goal of this paper is to come to a comprehensive behaviour change model for advance care planning based on the Stages of Change model. First, we describe the Stages of Change model. Subsequently, we outline three issues that arise when applying the Stages of Change model to advance care planning. Based on current literature and original data on advance care planning in a nationwide sample of older people, we will propose the Advance Care Planning–Applied Stages of Change model (ACP-ASC model). Last, we will discuss further considerations and implications for practice and research.

## The Transtheoretical model: Stages of Change

The Transtheoretical model (TTM) by Prochaska and DiClemente^9–11^ has been proven helpful for many different health behaviours, including smoking cessation^28^ and cancer screening.^29^ It includes several constructs, including individuals’ current behavioural status and intention to change (‘Stages of Change’), activities used to progress through these stages (‘processes of change’), and the level of confidence in (‘self-efficacy’) and arguments for and against (‘decisional balance’) behaviour change.^9–11^ The Stages of Change model includes five stages, ranging from not thinking about change (precontemplation) to sustaining behaviour (maintenance; Figure 1).^9^ In addition, a cyclic component of regression is recognized, that is, the *process of relapse*, in which people return from action or maintenance to an earlier stage.^9–11^

## Issues with applying the stages of change to advance care planning

### Issue 1: Advance care planning includes not one but (up to) three types of actions

The action stage of the Stages of Change model is defined as having made behaviour change, in this case being engaged in advance care planning. However, ‘engagement in advance care planning’ can mean different things. It may involve discussing preferences with family/friends, but can also mean discussing preferences with healthcare professionals and/or documentation. We argue that the proposed ACP-ASC model should include these three distinct advance care planning actions, each to be addressed individually within the action stage.

#### Insights from literature

The European Association of Palliative Care (EAPC) supported consensus definition of advance care planning distinguishes three types of advance care planning actions: discussing preferences with family or friends, with healthcare professionals, and, where appropriate, documentation.^3^ The Delphi panel for the consensus definition reported advance care planning discussions with family/friends and with healthcare professionals as ‘central elements’ of the advance care planning process and stated that ‘advance care planning *can* include the documentation of preferences and appointment of a proxy decision maker’.^3^ This reflects the contemporary understanding of advance care planning, emphasizing its role as an ongoing communication process.^1–3^ The three types of advance care planning actions represent methods for conceptualizing and expressing wishes for future care, rather than being goals themselves.

The type, content, legal status, and use of documentation of future preferences varies considerably across countries, as does the terminology. For example, in the UK, a distinction is made between advance decisions (i.e. binding refusals of specific treatments, such as Do Not Attempt Cardiopulmonary Resuscitation (DNACPR) decisions); advance statements (i.e. preferences for future care); and lasting powers of attorney (i.e. appointment of surrogate decision-makers).^30^ In the United States, documents include advance directives (e.g. living wills, including preferences for, and refusals of, specific treatments); durable power of attorney for healthcare (appointment of surrogate decision-makers); and Portable Orders for Life-Sustaining Treatment (POLST), which are intended for individuals with advanced, life-limiting conditions.^31^ Additionally, healthcare professionals may document patients’ future preferences, discussed in advance care planning discussions, within the medical record.

For a behaviour change model to accommodate the advance care planning process, it is important to distinguish these three types of advance care planning actions for two reasons. First, each type of advance care planning action serves a different purpose. For instance, advance care planning discussions help individuals to conceptualize their personal values, whereas documentation serves distinct legal purposes.^32^ Recognizing the distinct purposes of each action underscores the idea that all should ideally be undertaken and promoted, as they are complementary.

Second, beyond general barriers to advance care planning, such as avoidance of acknowledging death and dying,^33^ each type of advance care planning action presents unique barriers. For instance, absence of close family/friends,^34^ family issues,^34–38^ concerns about burdening family/friends,^36,38,39^ and discomfort among family/friends,^12,34,39^ may prevent people from engaging in advance care planning discussions with family/friends, but not necessarily from engaging in other advance care planning actions. Whereas lack of trust and negative perceptions of healthcare professionals’ time for and interest in advance care planning hamper advance care planning discussions with healthcare professionals.^37,40^ By distinguishing the three types of advance care planning actions, the model prompts researchers and healthcare professionals to be attentive to the advance care planning actions’ unique purposes and barriers within the advance care planning process.

Concordantly, Sudore et al.^23^ specified the action stage with three types of advance care planning actions in a fixed order. Alternatively, Fried et al.^18^ separated the advance care planning actions in three separate behaviour change models. This separation implies that each advance care planning action is an independent goal and overlooks the interconnected and complementary nature of these actions within the advance care planning process.

#### Insights from a nationwide sample

**Figure 2.**
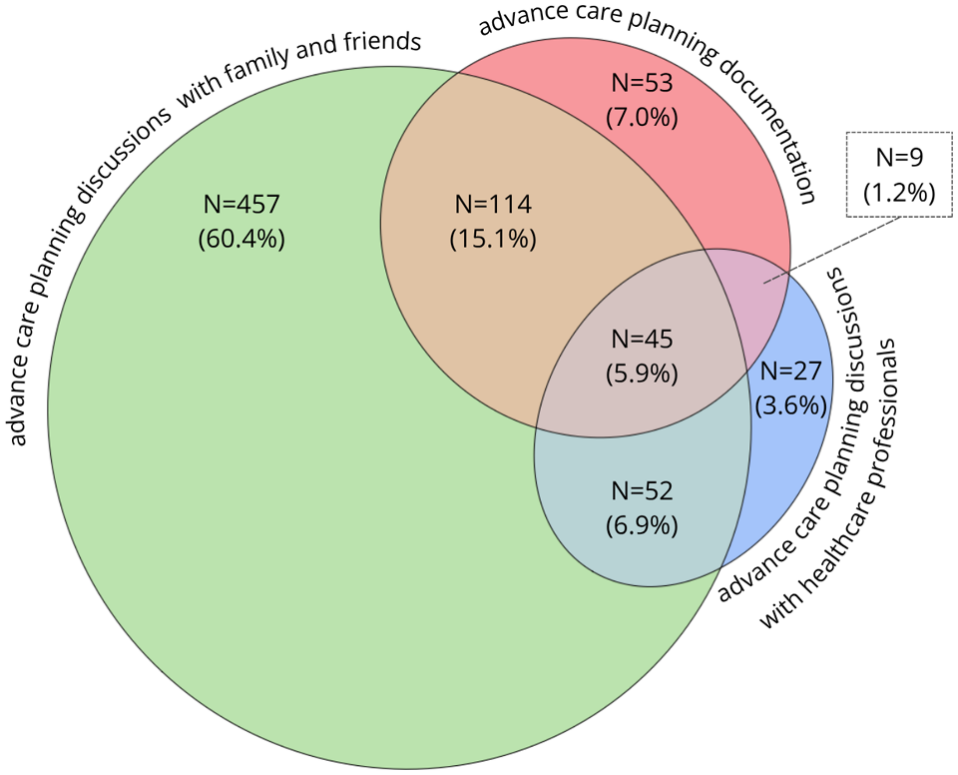
Engagement in advance care planning action(s) (N = 757).

To better understand the advance care planning process, we performed analyses in a nationwide sample of older people in the Netherlands. The data source, population, methods and respondents’ characteristics are described in Supplemental File A. Of the 1436 participants, 757 did engage in any advance care planning action (61.2%) and 479 did not (38.8%). Figure 2 presents the (combinations of) advance care planning action(s) participants engaged in. All possible combinations of advance care planning actions were seen, only 5.9% engaged in all three advance care planning actions. These findings suggest that characterizing engagement in advance care planning in binary terms is not accurate.

#### Proposal: The action stage includes three distinct advance care planning actions

The action stage of the ACP-ASC model is defined by presenting the three types of advance care planning actions individually: discussions with family/friends, discussions with healthcare professionals, and the documentation of preferences.

### Issue 2: The three advance care planning actions do not have a uniform order

We argue that there is no uniform order in how people (should) undertake the three types of advance care planning actions.

#### Insights from literature

Several studies (implicitly) assume an order in advance care planning actions. For instance, Westley and Briggs^25^ suggests that advance care planning discussions with family/friends and healthcare professionals are preparatory (preparation stage) to completion of advance care planning documentation (action stage). This is in line with the notion that was current at that time, but does not align with current insights. Further, Sudore et al.^23^ visualized the action stage as a sequential trajectory of advance care planning actions. While their model generally aligns with the original Stages of Change model, it specifies the action stage with the three types of advance care planning actions: starting with advance care planning discussions with family/friends, progressing to discussions with healthcare professionals, and concluding with documentation of an advance directive or proxy. However, Sudore et al. emphasize that advance care planning is an iterative process, where patients may revert to or skip stages depending on life circumstances or proactive healthcare professionals.^23^

Consistent with Sudore et al.’s notion, previous research found no evidence supporting a fixed order of advance care planning actions in the advance care planning process. Fried et al. reported ‘no evidence of a pattern of progression’ from one type of advance care planning action to another,^17,18^ later confirmed by Fleuren et al.^41^

Moreover, several practical arguments can be made against a uniform order of advance care planning actions. People may have reasons not to discuss preferences with family/friends (e.g. burdening family)^12,34–39^ or with healthcare professionals (e.g. lack of trust).^37,40^ Also, a healthcare professional might simply be the first to initiate advance care planning discussions before any advance care planning discussions with family/friends have taken place, for example due to deteriorating health or care transitions.^36,42^

#### Insights from a nationwide sample

Figure 2 presents the (combinations of) advance care planning action(s) participants engaged in. All different combinations of advance care planning actions were seen. If a specific order were true, certain combinations of advance care planning actions would be absent. These include, among others, the 15.1% of people who discussed wishes with family/friends (and not with healthcare professionals) and documented these, and the 7.0% of people who solely documented preferences without discussing these with anyone.

#### Proposal: The advance care planning actions do not have a uniform order

**Figure 3.**
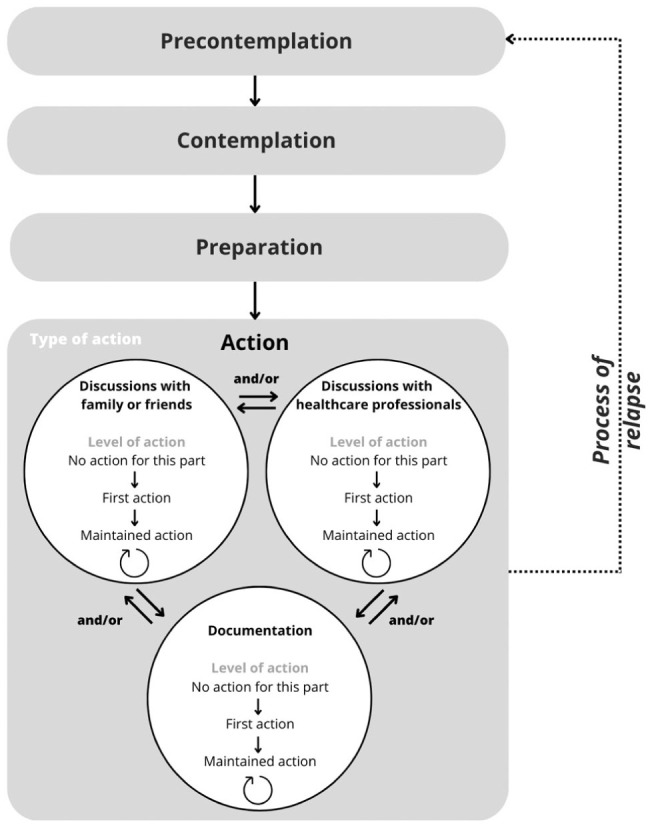
The Advance Care Planning–Applied Stages of Change model (ACP-ASC model).

In the ACP-ASC model, the distinct advance care planning actions in the action stage do not have a uniform order (Figure 3). This is visualized by arrows connecting the advance care planning actions in both directions, representing the simultaneous or sequential nature of the advance care planning actions individuals may engage in. Herewith, the three advance care planning actions provide different entry points for starting engagement in advance care planning.

### Issue 3: Maintenance is part of the ongoing advance care planning process

The maintenance stage is defined as the stage in which people have sustained a behaviour change over time.^9^ As advance care planning ideally is an ongoing process until death, we argue that maintenance is part of the advance care planning process itself, and should therefore not be regarded as a separate stage but rather as a level of action within the action stage.

#### Insights from literature

For smoking cessation, the maintenance of the behaviour change is quite obvious: people continue to refrain from smoking.^10^ According to the EAPC-supported consensus definition of advance care planning, people (ideally) reflect on their wishes and preferences regularly; advance care planning is an ongoing iterative process.^3^ Continued engagement in advance care planning discussions (and updating documentation) is thus central to the advance care planning process itself. This serves several purposes. Continued advance care planning discussions with family/friends and healthcare professionals support the process of conceptualization of personal norms and values. Further, in a significant minority of people preferences change over time,^43,44^ and continued discussions about preferences are needed.

#### Insights from a nationwide sample

The cross-sectional study design prevents us from drawing conclusions on repeated engagement in the three types of advance care planning actions.

#### Proposal: Maintenance is part of the ongoing advance care planning process

The re-engagement in one or (preferably) more advance care planning actions (i.e. maintenance) should be regarded as part of the action stage (Figure 3). The ACP-ASC model distinguishes three levels of action for each type of advance care planning action: no action (no action undertaken for this part), first action (first time the action is undertaken), and maintained action (regularly undertaken action). The latter substitutes the separate maintenance stage as formulated in the original TTM. With the incorporation of maintenance in the action stage, the process of relapse is redefined as regression from the action to the precontemplation stage, true when individuals have previously engaged in one or more advance care planning actions but have lost all commitment to the advance care planning process.

## The Advance Care Planning–Applied Stages of Change model (ACP-ASC model)

The ACP-ASC model (pronounced ‘ACP-ASK model’) as proposed by us in response to the described issues is presented in Figure 3. The terminology and operationalization are described in Table 1. For additional guidance in applying the ACP-ASC model, it has been applied to three hypothetical cases in Table 2.

**Table 1.** Operationalization and terminology of the ACP-ASC model.

**Terminology**
*Stage*: The current behaviour and intention to change according to the construct ‘Stages of Change’, i.e. precontemplation, contemplation, preparation, action (and maintenance in the original model).
*Type of action [new concept]*: The three types of advance care planning actions, i.e. advance care planning discussions with family/friends, advance care planning discussions with healthcare professionals, and documentation. The type of advance care planning actions represent methods for engaging in advance care planning, rather than goals in themselves.
*Level of action [new concept]*: The degree of action for each type of advance care planning action, i.e. no action, first action, and maintained action.
**Operationalization**
*Precontemplation*: Someone is not thinking about engaging in advance care planning. They might lack awareness of advance care planning or have no desire to engage.
*Contemplation*: Someone is thinking about engaging in advance care planning. They might orientate on advance care planning’s relevance for them.
*Preparation*: Someone is committing to engage in advance care planning in the near future. They may have taken some preparatory steps, such as looking up information on resuscitation or making an appointment with a healthcare professional to discuss preferences.
*Action*: Someone has engaged in advance care planning. They have undertaken, at minimum, one of the three types of advance care planning actions, either for the first time (first action) or regularly (maintained action).
*Process of relapse*: Someone has relapsed (specifically from the action to the precontemplation stage) when they have undertaken one or more advance care planning actions in the past, but have subsequently lost all commitment to or trust in the advance care planning process as a whole and express no interest in undertaking any further actions in the future. This may occur, for example, as a result of a negative experience with advance care planning.

**Table 2.** Hypothetical cases.

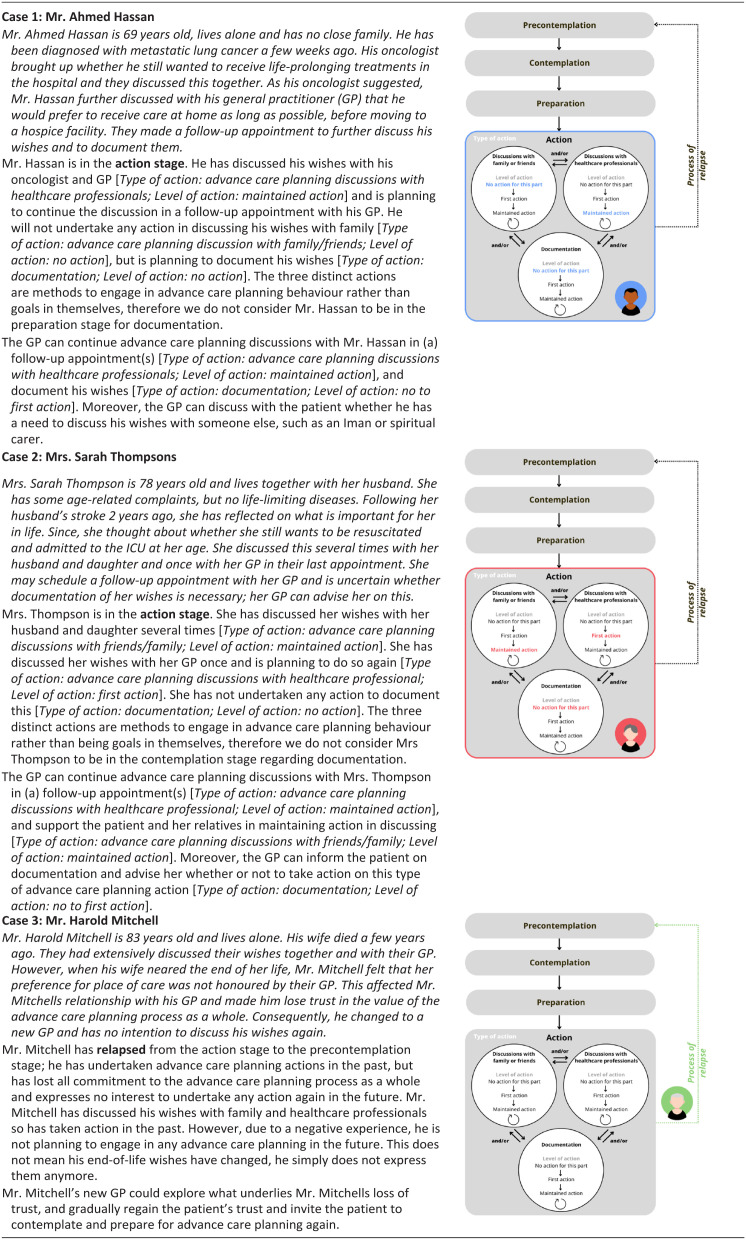

Bold formattings is to highlight the corresponding stage of the ACP-ASC model in the hypothetical case.

## Closing comments and future directions

This paper describes the Advance Care Planning–Applied Stages of Change model (ACP-ASC model), which integrates the different types of advance care planning actions in a single behaviour change model and reflects todays understanding of advance care planning as iterative and ongoing process. We have argued that advance care planning includes three types of advance care planning actions without a uniform order, and that continued engagement in advance care planning (i.e. maintenance) is part of the advance care planning process itself. Therefore, the ACP-ASC model specifies the action stage with the three types of advance care planning actions (i.e. discussions with family/friends, discussions with healthcare professionals, and documentation) and the level of action (i.e. no action, first action, and maintained action) for each type of action. The ACP-ASC model can be helpful for (intervention) research and healthcare practice as it defines and distinguishes the type(s) and level(s) of advance care planning actions.

### Further considerations

Advance care planning encompasses a broad range of topics, including resuscitation, hospitalization, preferred place of care, and, in some jurisdictions, euthanasia. These topics may be addressed based on individual relevance or interest at the time. Discussions can revisit previously explored topics or introduce new ones, meaning that re-engagement in advance care planning does not necessarily reflect the breadth of topics discussed. Also, individuals may engage in the same type of advance care planning actions with different people, such as various loved ones or healthcare professionals (e.g. oncologist, nurse, general practitioner). Further, the ACP-ASC model currently limits discussions with professionals to healthcare practice, aligning with the current definition of advance care planning.^3^ However, future models might broaden this to include professionals from the social or spiritual domain. The diversity of advance care planning topics and the range of individuals involved highlight the ongoing, iterative nature of the advance care planning process. Conceptually, the ACP-ASC model does not differentiate between these variations.

In the original Stages of Change, specific time frames were assigned to each stage. We chose not to incorporate this aspect, as categorization into stages of advance care planning behaviour should be solely based on qualitative information, namely the level of intention and/or preparation to change, rather than on specific time frames, as previously argued.^45^ Furthermore, the original Stages of Change allows relapse from action and maintenance to any preceding stage.^9–11^ However, in advance care planning, relapse is inherently complex and requires a more precise definition. In the ACP-ASC model, relapse is defined specifically as regression from action to pre-contemplation, indicating that individuals who were previously engaged in advance care planning have lost all commitment to or trust in the advance care planning process as a whole. Although the practical occurrence of this regression remains unknown, it can occur; for example, when a relative’s previously discussed wishes regarding preferred place of care cannot be honoured due to practical constraints at that time (see Table 2. Hypothetical cases. Case 3). Such experiences can undermine trust in the advance care planning process and shift the decisional balance, leading to relapse.^9–11^ By contrast, the revision of wishes is regarded as an integral aspect of the advance care planning process and does not constitute relapse.

Last, the ACP-ASC model deliberately distinguishes first action and maintained action (i.e. levels of action). This explicit distinction highlights the need for research into trajectories and patterns of re-engagement (maintenance) in advance care planning, which remains limited. Moreover, many interventions still focus on initiating advance care planning rather than promoting continued advance care planning engagement. A systematic review found that only 4% of advance care planning interventions for patients with cancer targeted the maintenance stage, whereas 48%–92% focussed on other stages.^8^ Incorporating the levels of action into the model encourages healthcare professionals to consider not only whether a patient has ever engaged in advance care planning, but whether they do so regularly.

### Implications for practice

**Figure 4.**
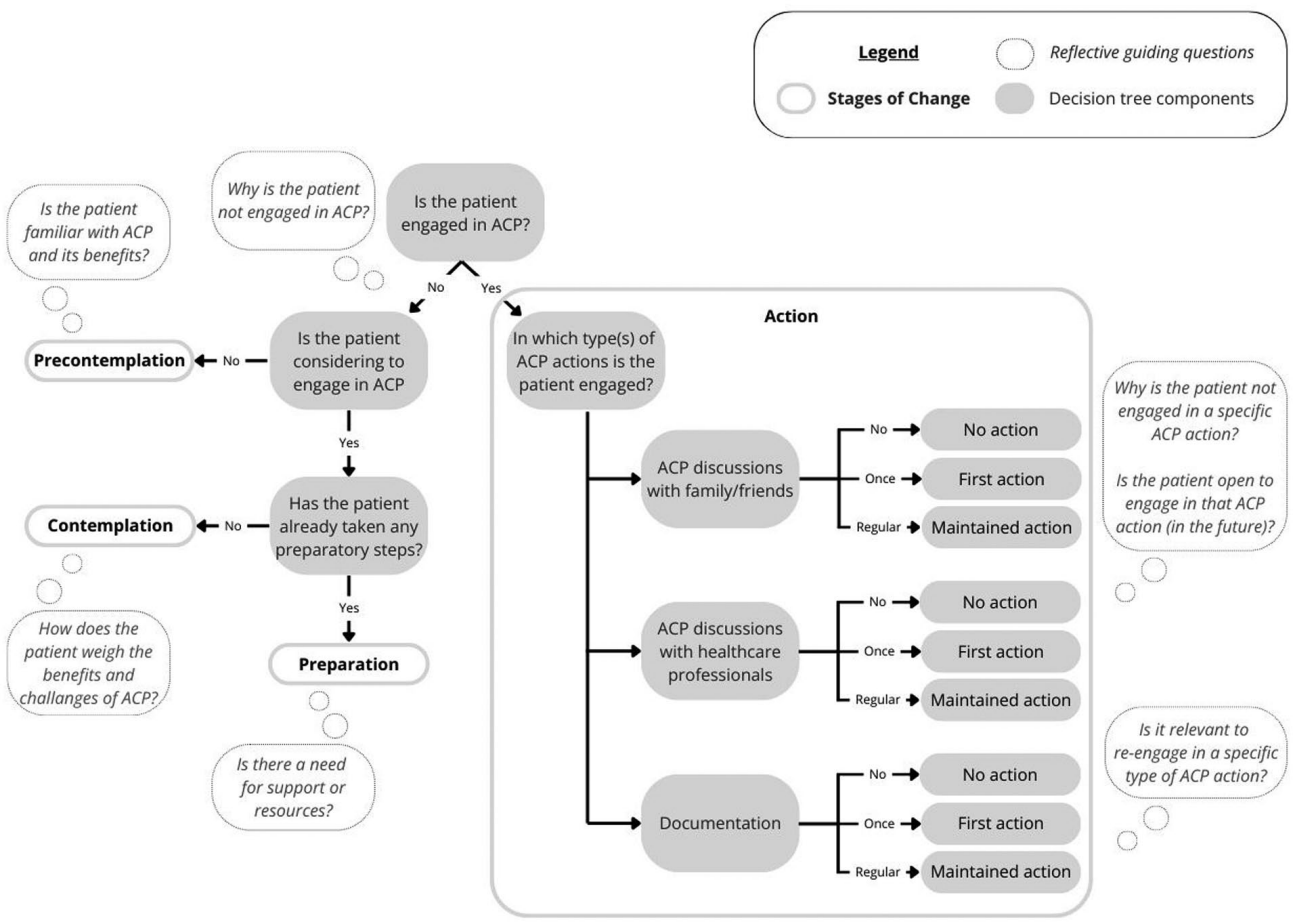
Decision tree with examples of reflective guiding questions to explore patient’s engagement in advance care planning.

The ACP-ASC model can be helpful for healthcare professionals as it offers guidance to explore patient’s engagement in advance care planning. Ideally, advance care planning is an ongoing process involving regular engagement, in which all actions are undertaken and promoted as complementary. If their patient is not engaged in advance care planning, it encourages exploration of the patient’s readiness to engage and consideration of whether additional support or resources are needed. If the patient is engaged in advance care planning, professionals could examine which types of advance care planning actions are being undertaken and whether done so regularly. Additionally, they may reflect on why a patient is not engaged in a specific type of advance care planning action and whether that would be beneficial in the future, whether support is needed, and whether re-engagement in specific types of advance care planning actions is relevant (e.g. to re-visit or expand on advance care planning topics). An example of a decision tree with reflective guiding questions is presented in Figure 4.

Furthermore, the ACP-ASC model enables development of advance care planning interventions matched to specific Stages of Change. Stage-matched interventions increase acceptability and effectiveness, thereby promoting behaviour change.^8,27^ The 10 processes of change identified by Prochaska and DiClemente^9–11^ offer strategies for overcoming barriers to behaviour (change) across stages. For instance, consciousness raising can address a lack of knowledge, a common barrier,^33^ and can especially support progression from (pre)contemplation.^8^ Intervention studies should specify targeted stage(s) of change and processes of change employed. Future implementation of the model should include thorough evaluation of its utility and effectiveness.

### Recommendations for future research

The ACP-ASC model provides directions for future research. Considering the lack of evidence for a uniform order in advance care planning actions, longitudinal research could provide insight in common entry points of advance care planning engagement and whether these are different for specific groups of individuals (e.g. age or patient groups). Further, in-depth qualitative research could explore why and how individuals (start to) engage in specific advance care planning actions.

Research on the maintenance of advance care planning is scarce. We could not illustrate the maintenance of advance care planning as our study lacked longitudinal data. Future longitudinal research could offer insight into patterns in and trajectories of the advance care planning process. Furthermore, qualitative research could add to the understanding of thought processes underlying (the lack of) regular engagement in advance care planning. Lastly, it remains unknown both how the process of relapse in advance care planning occurs in practice and how prevalent this is. Further research is warranted to explore this process.

### Strengths and limitations

A strength is that the ACP-ASC model builds on the widely accepted Stages of Change model and centres around the EAPC-supported consensus definition of advance care planning. It is based on both current literature and empirical data for illustrative purposes. In addition, the ACP-ASC model was conceptualized in thorough and constructive discussions in a multidisciplinary team of researchers with backgrounds in health sciences, psychology, medicine, ethics and sociology, and included healthcare professionals (i.e. practising general practitioner and former nurse).

One limitation of this paper is the absence of longitudinal data. The cross-sectional nature of the data prevents the authors from drawing conclusions about the order of occurrence, particularly regarding the maintenance of advance care planning. Additionally, the data used in this study included a representative sample of older adults, however conclusions about the general population should be drawn with caution. The data was used primarily for illustrative purposes. Finally, it is important to note that a conceptual model is a simplification of reality and cannot capture all the nuances of the advance care planning process. This is, for example, evident in the process of relapse, which is inherently complex in the context of advance care planning and for which it remains unknown how it occurs in practice.

## Conclusion

This paper outlines a comprehensive behaviour change model for advance care planning based on the Stages of Change model of the Transtheoretical model: the ACP-ASC model. It provides further insight in the advance care planning process and targets specific points for the development and evaluation of interventions.

## Supplemental Material

sj-docx-1-pmj-10.1177_02692163261431148 – Supplemental material for The ACP-ASC model: A comprehensive behaviour change model for advance care planning based on the Stages of Change model Supplemental material, sj-docx-1-pmj-10.1177_02692163261431148 for The ACP-ASC model: A comprehensive behaviour change model for advance care planning based on the Stages of Change model by Tessa D. Bergman, Bregje D. Onwuteaka-Philipsen, Eva E. Bolt, H. Roeline W. Pasman and Annicka G. M. van der Plas in Palliative Medicine
