# Supplementary material for: The ACP-ASC model: A comprehensive behaviour change model for advance care planning based on the Stages of Change model
Source: Palliat Med. 2026 Mar 29;40(6):868–78. doi: 10.1177/02692163261431148 (PMC13221577; doi:10.1177/02692163261431148)
Supplement: sj-docx-1-pmj-10.1177_02692163261431148 – Supplemental material for The ACP-ASC model: A comprehensive behaviour change model for advance care planning based on the Stages of Change model [file sj-docx-1-pmj-10.1177_02692163261431148.docx]

**Supplementary file A**

*Data source and methods*

A full description of the study design, data collection, participants, and characteristics of respondents has previously been published by Bergman et al. (2023, 2025).

A cross-sectional study was conducted with the use of data of the Longitudinal Internet Studies for the Social sciences (LISS) administered by Centerdata (Tilburg University, The Netherlands). The LISS panel participates in a yearly longitudinal questionnaire, covering a large variety of domains including demographics (Scherpenzeel, 2009, 2018), and monthly questionnaires on specific topics. In September 2020, a questionnaire about palliative care, specifically designed by Amsterdam University Medical Center (Amsterdam UMC), was send to a randomly drawn sample of 1.333 individuals aged 65 and older from the LISS panel. This resulted in 1,242 individuals (response rate of 93.2%). Half of the respondents were female (50.8%). The majority of respondents were aged either 65‒70 years (32.7%) or 70‒75 years (33.3%). Most respondents were not religious (63%) and had a low level of education (40.4%). A minority perceived their health status as poor (21.5%).

Respondents were asked whether they had discussed wishes with family/friends, discussed wishes with a healthcare professional, and/or had written down wishes about the following topics: whether or not they want / can continue to live at home, would like to go to hospital, would like to be admitted to a nursing home, want to be resuscitated, which treatments they would and would not want any more in certain circumstances, would want euthanasia in certain circumstances, and who could make medical decisions for them if they are no longer able to do so themselves. For the purpose of analyses, respondents were categorised in the following (not mutually exclusive) categories of ACP behaviour: people who [A] discussed one or more end-of-life topics with family/friends, [B] discussed one or more end-of-life topics with a healthcare professional, and [C] had documented wishes regarding end-of-life care. Descriptive statistics were conducted on these ACP behaviours using SPSS IBM 28.

**Literature**

Bergman T. D., van der Plas, A. G. M., Pasman, H. R. W., & Onwuteaka-Philipsen, B. D. (2023). Awareness and Actual Knowledge of Palliative Care Among Older People: A Dutch National Survey. *J Pain Symptom Manage, 66*(3), 193–202.e192. doi:10.1016/j.jpainsymman.2023.05.005

Bergman Tessa D., van der Plas, A. G. M., Onwuteaka-Philipsen, B. D., & Pasman, H. R. W. (2025). Two-thirds of older people is interested in information meetings on end-of life care to stimulate advance care planning: a national survey. *BMC geriatrics, 25*(1), 574. doi:10.1186/s12877-025-06231-x

Scherpenzeel A. (2009). Start of the LISS panel: Sample and recruitment of a probability-based Internet panel. *CentERdata, Tilburg*.

Scherpenzeel A. (2018). ““True” Longitudinal and Probability-Based Internet Panels: Evidence From the Netherlands. In *Social and behavioral research and the Internet* (pp. 77–104): Routledge.
